# Ecoinformatics Can Reveal Yield Gaps Associated with Crop-Pest Interactions: A Proof-of-Concept

**DOI:** 10.1371/journal.pone.0080518

**Published:** 2013-11-15

**Authors:** Jay A. Rosenheim, Matthew H. Meisner

**Affiliations:** Department of Entomology and Nematology, University of California Davis, Davis, California, United States of America; Federal University of Viçosa, Brazil

## Abstract

Farmers and private consultants execute a vast, decentralized data collection effort with each cropping cycle, as they gather pest density data to make real-time pest management decisions. Here we present a proof of concept for an ecoinformatics approach to pest management research, which attempts to harness these data to answer questions about pest-crop interactions. The impact of herbivory by *Lygus hesperus* on cotton is explored as a case study. Consultant-derived data satisfied a ‘positive control’ test for data quality by clearly resolving the expected negative relationship between *L. hesperus* density and retention of flower buds. The enhanced statistical power afforded by the large ecoinformatics dataset revealed an early-season window of crop sensitivity, during which *L. hesperus* densities as low as 1-2 per sample were associated with yield loss. In contrast, during the mid-season insecticide use by farmers was often unnecessary, as cotton compensated fully for moderate *L. hesperus* densities. Because the dataset emerged from the commercial production setting, it also revealed the limited degree to which farmers were willing to delay crop harvest to provide opportunities for compensatory fruiting. Observational approaches to pest management research have strengths and weaknesses that complement those of traditional, experimental approaches; combining these methods can contribute to enhanced agricultural productivity.

## Introduction

A growing world population will necessitate a substantial increase in global agricultural production [1]. Part of this increase can come from more effective suppression of damaging pest populations [2]. At the same time, indiscriminate use of pesticides is undesirable, because of negative consequences for human and environmental health, and because of the potential to accelerate the evolution of resistance and disrupt ecosystem services contributed by natural enemies [3]. Thus, we need an efficient means of distinguishing between conditions under which pests cause yield shortfalls versus conditions under which crop plants can compensate for herbivory, rendering pesticide applications or other pest control interventions unnecessary.

 Researchers have traditionally relied on manipulative experiments to identify conditions under which pests depress yield. Although experimentation will continue to be crucial, alternatives to experimentation that have hitherto received little attention may play a complementary role. In particular, observational approaches may be useful in providing greater statistical power for detecting the small, but economically important, yield losses that drive farmer decision-making [4]. Key yield losses are often small in magnitude for two reasons. First, most crops are impacted by many pests, including diverse arthropod, nematode, pathogen, and weed populations, each of which may generate small effects, but which can, collectively, depress yield substantially. Because pests are generally managed individually or in small groups rather than in aggregate, we need to resolve individual effects. Second, to minimize unnecessary pesticide applications, researchers must be able to resolve the yield loss decision point at which a profit maximizing farmer will opt to apply a pesticide to protect crop yield (the “economic injury level”; [5]). Because the cost of a pesticide application is often small relative to the value of the crop, this decision point is often reached when only a tiny loss of yield (ca. 2%) is threatened; experiments generally do not successfully resolve effects of this size [4]. In some cases, researchers have formed consortia to replicate experiments extensively in space and time, creating composite data sets that enhance statistical power. This approach has had notable success [6-8], but can only be used when substantial research resources are available, and as such can be used only for the most important pests in developed countries and perhaps only very infrequently in developing countries.

 Ecoinformatics, which involves mining pre-existing data sources to generate large, observational data sets, holds promise as a lower-cost means of generating higher-resolution insights into pest impacts on crop yield. Farmers monitor pest densities in their crops to guide real-time pest management decision-making. This vast, decentralized data collection effort can be combined with farmer data on realized crop yields to generate large observational datasets quantifying crop-pest interactions.

 Here we present a proof of concept for the application of ecoinformatics to the study of pest impacts on crop yield. A long-standing controversy surrounds the possible impact of *Lygus hesperus* Knight (Heteroptera: Miridae) on cotton production in the Western United States. *L. hesperus* prefer to feed on young flower buds on cotton, and plants may respond by abscising damaged buds [9-11]). However, decades of experimentation, focused nearly exclusively on mid-season (July) *L. hesperus* herbivory, have resolved yield effects only at high pest densities (>8 insects per standard sample of 50 sweeps; [12-17]). Cotton plants exhibit plasticity in growth; thus, plants can potentially compensate for herbivory that causes early flower bud abscission by increasing the subsequent production of ‘replacement’ flower buds, although this may delay fruit maturation and crop harvest [15,18,19]. Farmers, however, remain skeptical of university recommendations for high *L. hesperus* treatment thresholds. Farmers may be unwilling to extend the cotton growing season into the fall, when a variety of crop risks may be present, and generally apply insecticides at much lower densities (2-4 *L. hesperus* per sample). Furthermore, farmers generally use a sliding threshold: earlier in the growing season (pre-flowering; roughly June in California), farmers often use a threshold near 2 bugs per sample, whereas later in the growing season (roughly July), farmers often use a threshold near 4 bugs per sample. Farmers rarely suppress *L. hesperus* after early August, when flower buds have diminished chances of developing to mature fruit prior to harvest. 

 A renewed focus on *L. hesperus* is timely for two reasons. First, a new cotton species*, Gossypium barbadense* L. (“Pima cotton”), has replaced *Gossypium hirsutum* L. (“upland cotton”) as the dominant species cultivated in California, and it is not known if the impacts of *L. hesperus* on these two cotton species are similar. Second, the widespread adoption of transgenic cotton cultivars expressing endotoxin genes from *Bacillus thuringiensis* and the associated withdrawal of broad-spectrum insecticides targeting lepidopteran pests has resulted in the emergence of *Lygus* spp. and other hemipteran pests as primary threats in many of the world’s cotton producing regions [8,20,21].

 To build a proof of concept, we will first address a primary perceived obstacle to this approach, namely that farmer- or consultant-derived data would be too heterogeneous or of insufficient basic quality to reveal signals of herbivore impact on crop performance. Although *L. hesperus* impact on cotton yield is controversial, there is no question that *L. hesperus* contributes to flower bud abscission [9-11,15,22,23]. We will therefore ask if a farmer-derived dataset can resolve this expected effect as a positive control for data quality. We will then use the farmer-derived data set to evaluate the possibility that crop sensitivity varies across the growing season by exploring associations between early- (June) and mid-season (July) herbivory by *L. hesperus* and (i) cotton yield and (ii) the time when farmers opt to terminate crop growth in preparation for harvest.

## Materials and Methods

The data set was built exclusively by collecting pre-existing data derived from observations of commercial cotton production in California’s San Joaquin Valley. Data streams included the following:

- *Lygus hesperus* densities. *L. hesperus* population densities were sampled by four independent pest control consulting firms, who are employed by farmers to provide pest monitoring services and control recommendations. The final data set included observations for 1432 cotton crops produced by 35 farms between 1997 and 2008. All consultants used the same standard sampling procedure: an approximately weekly series (typically 6-12) of sweep net samples, each comprising 50 swings of a sweep net across the top of the plant canopy. Successive samples were transformed into mean *L. hesperus* density estimates by calculating the area under the curve of *L. hesperus* density versus time, using linear interpolation between successive density estimates and dividing by the total duration of the sampling interval; this accounted for the sometimes uneven time intervals between successive samples. Counts reflect all motile stages combined, as not all consultants reported separate counts of nymphs and adults.
- Cotton yield. Farmers shared data on cotton lint yield (kg/ha) for years for which *L. hesperus* density estimates were available. Yield data were available for 1118 cotton crops.
- Historical cotton yield. Field to field variation in yield potential across the cotton growing region of the San Joaquin Valley (from Madera and Merced Counties in the north to Kern County in the south) can be substantial, due to differences in soil quality and local climate. Stable differences in farmer growing practices also contribute to between-field variation in yield. To control statistically for variable yield potential, we requested from farmers records on historical cotton yields for each of their fields as far back as their records permitted, or until 1990. With these historical data, the following quantities were calculated:
$\text{Expected yield deviation = }\sum_{i\neq f}^{}\frac{y_{i}-{\bar{y}}_{i}}{N},$

where *y*_*i*_ is the yield observed in a particular field during year *i*, ${\bar{y}}_{i}$, is the mean yield observed for year *i* across the entire San Joaquin Valley, as reported by the USDA (http://www.nass.usda.gov), and *N* is the number of years between 1990 and 2008, other than the focal year, year *f*, for which yield estimates were available for the cotton species in question; 

$$\text{Observed yield deviation = }y_{f}-{\bar{y}}_{f},$$

where *y*_*f*_ is the yield observed for a particular field-year for which *L. hesperus* density estimates were available and ${\bar{y}}_{f}$ is the mean yield observed for that year across the entire San Joaquin Valley; and finally:

Yield gap = Observed yield deviation - Expected yield return.

The yield gap was the response variable for analyses of yield effects.

- Flower bud retention. Pest management consultants and, in some cases, independent agronomy consultants provided estimates of flower bud retention, produced by scoring retention at the first position on fruiting branches located on the top five mainstem nodes of a sample of cotton plants.
- Pesticide applications. The dates and pest targets of all pesticide applications were obtained from consultants and the California Department of Pesticide Regulation’s on-line Pesticide Use Reporting system (http://www.cdpr.ca.gov/docs/pur/purmain.htm). Pesticide applications made at planting (i.e., aldicarb) provided data on planting date.
- Plant growth regulator applications. The plant growth regulator mepiquat chloride is used by farmers to shift the plant’s resource allocations from vegetative to reproductive growth, which may enhance yield [24]. Because *L. hesperus* herbivory can trigger increased use of mepiquat chloride [25], we obtained data on mepiquat chloride from consultants.
- Date of first application of chemical defoliant. Cotton must be defoliated prior to harvest. The date of first defoliant application was used as an indicator of the time when the farmer opted to terminate the crop. Defoliant application dates were obtained from pest control consultants and the California Department of Pesticide Regulation. To combine data across years, dates were expressed as [(date of first defoliant application) – (mean date of first defoliant application for the year in question)]; thus, positive numbers indicate application dates that were later than average. These data were used to quantify farmer willingness to extend the growing season in response to *L. hesperus* damage to allow for plant compensation.
- Weather. Temperature and precipitation have the potential to affect both cotton yield and *L. hesperus* populations. We obtained the mean monthly temperature and precipitation from weather stations in the San Joaquin Valley from the National Oceanic and Atmospheric Administration's National Climatic Data Center (http://www.ncdc.noaa.gov). For each record in the database, we used climatic data from the nearest weather station (mean ± SD distance to station, 13.1 ± 7.2 km) with available data for that year.

Data were managed in a relational database programmed in SQL Server and accessed using an interface designed by a private software developer (“*Cottonformatics*”, Ten2Eleven Business Solutions). 

### Statistical analysis

Both the empirical record and theoretical treatments of plant-animal interactions suggest that the relationship between herbivore densities and host plant performance will often be non-linear, with losses accelerating as plant damage increases [26-28]. *L. hesperus* impacts on cotton were therefore analyzed using a flexible non-linear regression method, generalized additive models (GAM), implemented using program mgcv 1.7-6 in R [29]. GAMs are attractive because they allow the data “to speak for themselves” in suggesting the form of the function, rather than imposing a particular model (linear, quadratic, etc.) on the data. GAMs also provide an objective means of avoiding the over-fitting of the data (i.e., fitting the noise instead of the underlying trends) through a process that penalizes excessive “wiggliness” of the resulting function, as quantified by the second derivative of the curve (generalized cross validation). To keep the analyses as transparent as possible, we opted to analyze quite simple models; three response variables (flower bud retention, yield gap, and the date of the first application of defoliant) were analyzed separately, and in all cases the models included main effects of farm, year, *Gossypium* species, and mean *L. hesperus* densities during specified time intervals. To model the date of the first defoliant application, we also included mean local monthly temperature and precipitation variables. The overall modeling approach was semi-parametric: only the *L. hesperus* density variables were smoothed, using non-parametric thin plate regression splines, with the remaining variables treated as traditional linear additive effects in the usual parametric fashion. All analyses employed a Gaussian distribution and the identity link function.

To explore the possibility that other factors, correlated with both *L. hesperus* densities and cotton yield, might create spurious associations between *L. hesperus* and cotton performance, we explored GAM models including other variables. Because not all variables were measured for all records, we could not build a single model with all factors considered simultaneously without sacrificing many data records. We therefore conducted a series of analyses to explore different sets of possible confounders. Although it is not possible to exclude the possibility of unmeasured confounding variables, we attempted to search diligently for possible hidden sources of causality. Because insecticides applied to control *L. hesperus* may trigger secondary pest outbreaks that could depress yield [30], we explored a model including the season-long number of insecticide applications that targeted *L. hesperus*. Because wet years are thought to be associated with both higher *L. hesperus* densities [31] and delayed planting, causing lower yields, we explored a model that included planting date, expressed as a deviation from the mean planting date for the year in question. We also explored a model that included mean local monthly temperature and precipitation values. Because *L. hesperus* densities may be lower in larger fields [32,33], and because larger fields may be correlated with agricultural intensification, and thus be associated with higher yields, we explored a model that included field size (ha). Because *L. hesperus* densities might be correlated with densities of other herbivores, including spider mites (*Tetranychus* spp.), aphids (*Aphis gossypii* Glover), whiteflies (*Bemisia tabaci* [Gennadius] and *Trialeurodes vaporariorum* [Westwood]), thrips (*Frankliniella occidentalis* [Pergande]), and Lepidopteran caterpillars (various, but primarily *Spodoptera* spp.), we explored a model that included the numbers of pesticide applications targeting each of these herbivores. Finally, because *L. hesperus* herbivory may change plant growth form, eliciting changes in farmer decisions to apply plant growth regulators (mepiquat chloride) or defoliants, each of which may influence yield [25], we explored a model including the number of these applications.

 An additional concern is that our GAM analyses might not isolate correctly the associations between June versus July *L. hesperus* populations and cotton yield if insect densities are strongly correlated in time. June and July *L. hesperus* densities were positively correlated across our full data set, but the association was not tight, perhaps in part due to insecticide use (Pearson’s correlation, *R* ^2^ = 0.29, *N* = 1432, P < 0.0001). To determine if this modest correlation might be generating interpretational errors, we performed stepwise multiple linear regression analyses examining the effects of June, July, and June x July *L. hesperus* densities on yield. By comparing different orders of variable inclusion, we evaluated the possibility that *L. hesperus* densities during June might mask important correlates of later herbivory. This analysis also allowed us to test for an interaction between June and July *L. hesperus* (GAMs assume additivity).

## Results

### A positive control for data quality

**Figure 1:**
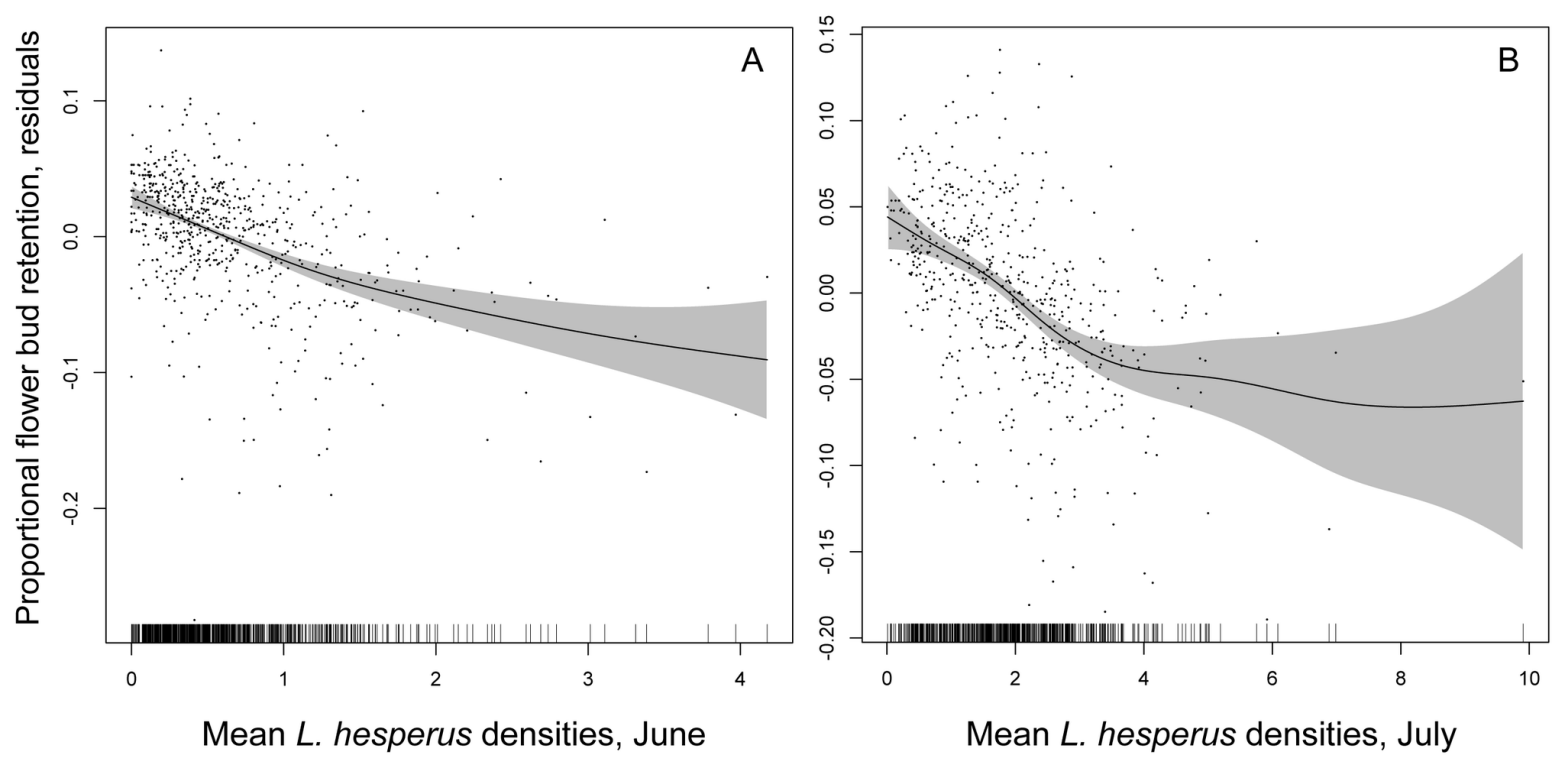
Flower bud retention and *L*. *hesperus* density. Association between the proportion of cotton flower buds retained and mean *L*. *hesperus* densities observed during (A) June, and (B) July. Retention values are residuals after controlling statistically for effects of farm, year, and cotton species. The solid lines are the smooths from the GAM models, and the shaded regions are the 95% confidence intervals.

The quality of the farmer-derived data was sufficiently high that the expected negative impact of *L. hesperus* on flower bud retention was clearly revealed (Figure 1). During June, the full GAM model explained 59.5% of the deviance (Table 1), roughly half of which was attributable to *L. hesperus* (a model including only *L. hesperus* densities explained 30.5% of the deviance). In July the full model similarly explained 66% of the deviance (Table 2), but only a quarter of this was now attributable to *L. hesperus* (a model including only *L. hesperus* explained 16.4% of the deviance). Thus, the ecoinformatics data set passed this initial “positive control” test. The flower bud retention data furthermore suggested that the underlying damage generated by *L. hesperus* was similar in June and July; in both cases, flower bud retention dropped by ca. 10% as *L. hesperus* densities increased to ca. 4 insects/sample.

**Table 1:** Generalized additive model of factors associated with early-season (June) retention of flower buds by cotton.

Term	df	*F*	*P*
Farm	25	7.56	<1x10^-15^
Year	11	15.00	<1x10^-15^
*Gossypium* species	2	1.48	0.23
June *L. hesperus* density	2.37	27.45	<1x10^-15^

Deviance explained = 59.5%, *N* = 656

**Table 2:** Generalized additive model of factors associated with mid-season (July) retention of flower buds by cotton.

Term	df	*F*	*P*
Farm	24	9.61	<1x10^-15^
Year	11	27.02	<1x10^-15^
*Gossypium* species	2	5.76	0.003
July *L. hesperus* density	4.21	13.3	1.1x10^-12^

Deviance explained = 66.0%, *N* = 561

### *Lygus hesperus* densities and *Gossypium* yield

**Figure 2:**
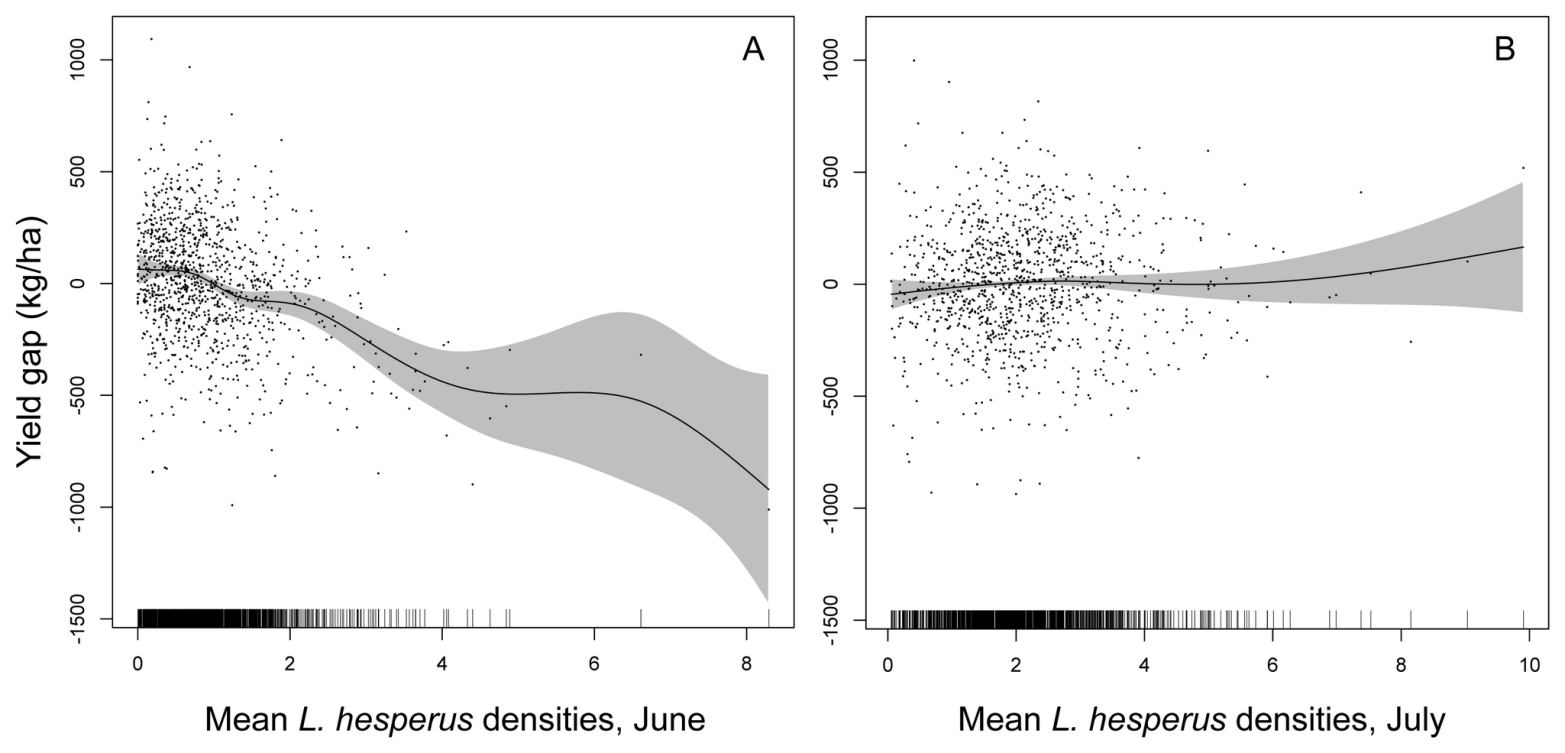
Cotton yield gap and *L*. *hesperus* density. Association between the cotton yield gap (difference between the observed and expected yield, after controlling for annual fluctuations in valley-wide yield) and mean *L*. *hesperus* densities observed during (A) June, and (B) July. The solid lines are the smooths from the GAM models, and the shaded regions are the 95% confidence intervals.

**Figure 3:**
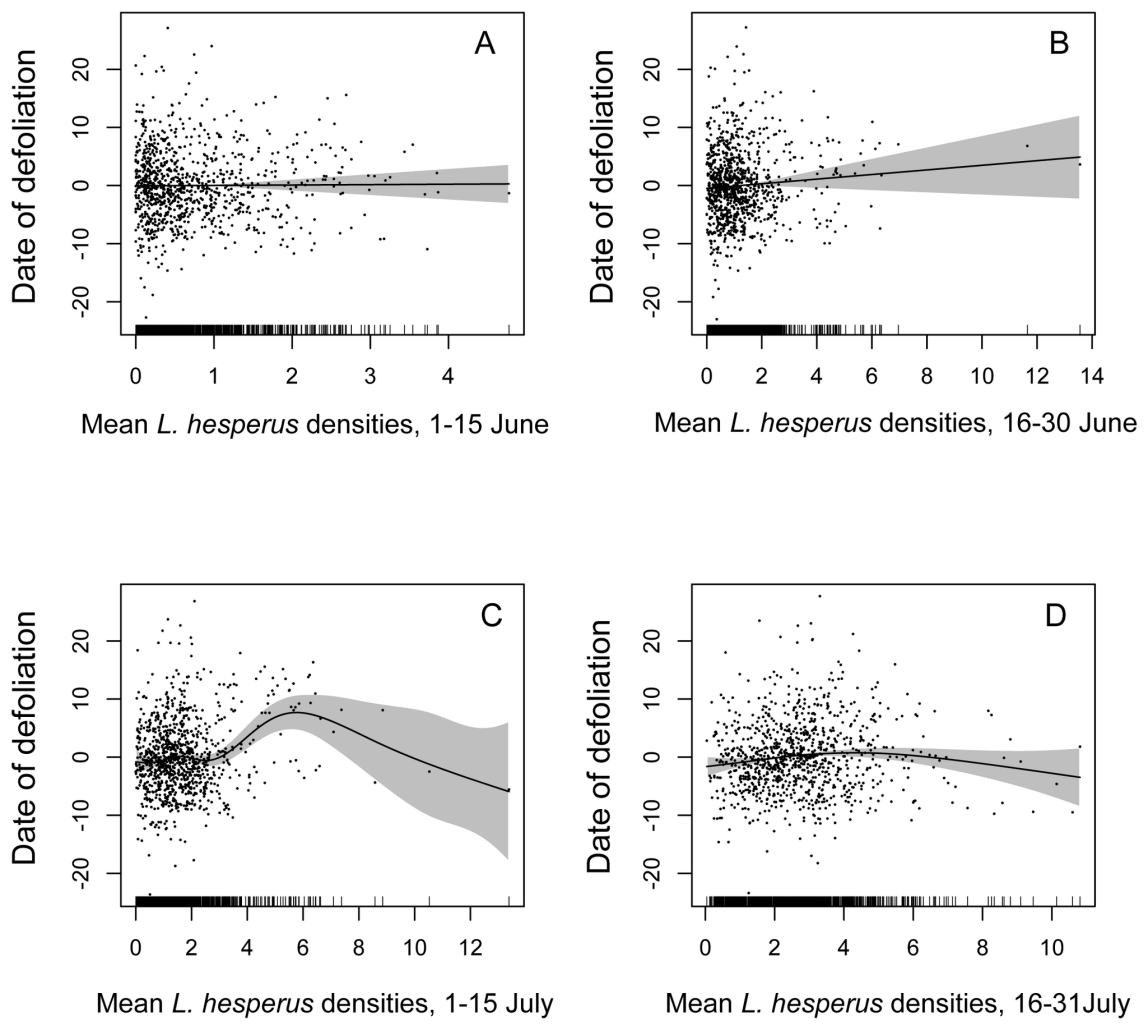
Crop termination and *L*. *hesperus* density. Association between the date of the first application of a defoliant to the cotton crop (expressed as a deviation from the year’s mean date of initial defoliant treatment) and mean *L*. *hesperus* densities observed during (A) 1-15 June, (B) 16-30 June, (C) 1-15 July, and (D) 16-31 July. The solid lines are the smooths from the GAM models, and the shaded regions are the 95% confidence intervals. Positive values for the date of defoliation indicate dates later than the mean for that year. Note that because the GAM penalizes the second derivative of the fitted curve, the model collapses to linearity when evidence for non-linearity is sufficiently weak, as is seen in panel (B). See Table S2 for the GAM summary.

 The ecoinformatics data set revealed an association between early-season (June) *L. hesperus* herbivory and depressed cotton yield that is expressed even at very low herbivore densities (1-2 *L. hesperus* per sweep sample; Table 3, Figure 2). This result did not rely on the highest *L. hesperus* density observations; repeating the analysis excluding the records (*n* = 88) with mean June *L. hesperus* densities >2.0 revealed the same significant yield decline associated with June herbivory (df = 2.6, *F* = 6.13, *P* = 0.0002). In contrast, the data revealed no evidence for any association between *L. hesperus* and yield later during the fruiting period (July), across the full range of *L. hesperus* densities represented in the data set (0-10 *L. hesperus* per sample). Repeating the analysis with the growing season broken into successive two-week periods suggested that cotton’s sensitive period may extend very weakly into the first half of July; thereafter, a non-significant trend towards increasing yield with increasing *L. hesperus* densities was observed (Table S1, Figure S1). Farmers delayed the first application of defoliant in fields that harbored moderate to heavy *L. hesperus* populations during 1-15 July (*P* = 0.00033; note that this effect may have reversed for the handful of fields that had the very highest *L. hesperus* densities). However, no such delays in crop defoliation were found to be associated with *L. hesperus* densities during June or 16-31 July (Figure 3, Table S2). Thus, during July, but not June, the plant appears to compensate strongly for the abscission of flower buds, perhaps aided by modest extensions of the crop’s growing period in response to early July herbivory. 

**Table 3:** Generalized additive model of factors associated with yield of cotton, *Gossypium* spp.

Term	df	*F*	*P*
Farm	35	2.44	8.3x10^-6^
Year	10	12.56	<1x10^-15^
*Gossypium* species	1	0.10	0.76
June *L. hesperus* density	6.39	9.24	7.7x10^-12^
July *L. hesperus* density	2.85	0.77	0.53

Deviance explained = 22.0%, *N* = 1118

As expected given the long list of factors that generate variation in cotton yield, densities of *L. hesperus* explained only a very small portion of the total observed yield variation (a model including only *L. hesperus* densities in June and July explained 7.8% of total deviance). Nevertheless, the large sample size (*N* = 1118 records) allowed this economically important effect to be resolved readily.

### Collinearity of June and July *L. hesperus* densities

Although our GAM analyses did support some non-linearities in the relationships between June *L. hesperus* and cotton yield, the overall trends were broadly linear. We therefore used stepwise multiple linear regression to examine if correlations between June and July *L. hesperus* densities might be masking effects of July herbivory. We forced Farm, Year, and *Gossypium* species into the model first. Using a threshold *P*-value to enter = 0.25 and a threshold *P*-value to remove = 0.10, June *L. hesperus* density was selected as the first and only variable to be added (*P* < 0.0001, *R* ^2^ = 0.065, ΔAICc = -73.4). July and June x July *L. hesperus* densities were not entered, despite the permissive threshold. If instead we forced July *L. hesperus* into the model as the first step, we obtained a modest improvement in fit (*P* = 0.0002, *R* ^2^ = 0.013, ΔAICc = -12.2), but the automated stepwise procedure then added June *L. hesperus* (*P* < 0.0001, *R* ^2^ = 0.066, ΔAICc = -60.6), and as the final step removed July *L. hesperus* (*P* = 0.24, *R* ^2^ = 0.065, ΔAICc = -0.6). Thus, we found no evidence that the modest correlation between June and July *L. hesperus* densities was hiding an underlying effect of July *L. hesperus* herbivory on crop yield.

### Searching for possible confounding variables

Statistical models including variables we deemed most viable as candidate confounders did not change the underlying relationship between *L. hesperus* densities and cotton yield. GAM models including the number of insecticide applications targeting *L. hesperus* (Table S3), the field’s planting date (Table S4), the field’s size (Table S5), the number of insecticide applications targeting other herbivores (Table S6), the number of applications of plant growth regulators or defoliants (Table S7), and mean local monthly temperature and precipitation (Table S8) all supported the same core inference: June *L. hesperus* were consistently associated with depressed cotton yield, whereas July *L. hesperus* were not.

## Discussion

The goal of this study was to develop a proof of concept for the application of ecoinformatics methods to quantifying pest impacts on crop yield, focusing on the impact of *L. hesperus* on cotton. *L. hesperus* was chosen as a test case, because, despite decades of experimentation, its optimal management remains controversial. Furthermore, the prospect of further exclusive reliance on experimentation was daunting: *L. hesperus* is so mobile that very large plots are required to maintain insect density manipulations, and secondary pest outbreaks are often associated with use of insecticides to manipulate *L. hesperus* densities, making interpretation of treatment effects difficult (e.g., [12,13]). The farmer- and consultant-derived data set passed an initial “positive control” test by resolving the expected impact of *L. hesperus* on flower bud retention (Figure 1). *L. hesperus* explained 30.5% (June) and 16.4% (July) of the deviance in flower bud retention. These values are comparable to three earlier researcher-generated data sets that examined the correlation between *Lygus* spp. densities and flower bud abscission across commercial upland cotton fields (*r* ^2^ = 0.21 [7]; *r* ^2^ = 0.22-0.44 [9] Leigh, Kerby & Wynholds 1988]; and *r* ^2^ = 0.19 [data reanalyzed from [34]]). Although data quality and heterogeneity will always be important concerns in ecoinformatics studies, these results encourage the hope that consultant-derived data can support useful research inferences. 

### Yield gaps: evidence from modeling and experimentation

Our analyses sought to identify conditions under which *L. hesperus* might be associated with unrecognized yield losses. Indeed, farmers appear to be losing yield when cotton harbors even very low *L. hesperus* densities during June, when cotton plants are producing their first flower buds. 

Our confidence that the association between June *L. hesperus* populations and loss of yield reflects a direct causal relationship would be bolstered by complementary evidence from other sources. We adduce two such sources of supporting evidence. First, two simulation models of *Gossypium* growth and fruiting, each parameterized with observations from commercial cotton farming in California’s San Joaquin Valley, but otherwise quite different in structure, each predicted that *Gossypium* would be most sensitive to flower bud loss early during the reproductive period ([22,35]). Thus, there was a clear reason, *a priori*, to expect that June *L. hesperus* herbivory would depress *Gossypium* yield.

 Second, a careful reexamination of the published experimental literature reveals suggestive evidence that early (June) *L. hesperus* herbivory depresses cotton yield. To our knowledge, only once have researchers manipulated June *L. hesperus* densities without continuing the manipulations later in the growing season. A June-only manipulation of *L. hesperus* densities is important, because season-long suppression of *L. hesperus* with insecticides often triggers secondary pest outbreaks, making such experiments hard to interpret (e.g., [12,13]). Falcon et al. [13] in their ‘1969 Experiment’ implemented a low *L. hesperus* density treatment (“5 early”) by applying insecticides any time *L. hesperus* densities reached 5 per sweep sample during the earliest period of fruiting (<5 blooms/4 m of plants; roughly June, in some plots extending until July 8). The manipulation was then relaxed for the remainder of the growing season. Mean June *L. hesperus* density per sweep sample in the ‘5 early’ treatment plots was 1.48, substantially less than the 5.50 observed in the untreated control. To achieve these clear density differences during June, when nearly all *L. hesperus* are present as mobile, winged adults (86% adults in this experiment), required the use of very large plots: plot dimensions were 201 x 805 m. As a result, the level of replication was small (*n* = 4), and statistical power was limited. Nevertheless, the first cotton harvest revealed a statistically significant 10.7% yield loss in the untreated control compared to plots where *L. hesperus* densities were suppressed (*P* < 0.05). The second harvest was very small in comparison to the first harvest (producing just 6.1% more yield), and changed the picture only slightly: early *L. hesperus* were still associated with a 9.3% loss of yield. Nevertheless, inclusion of the second harvest resulted in the loss of the statistical significance of the yield gap (*P* > 0.05). The original authors discussed these highly suggestive trends, and called for further investigations of the yield gaps stemming from early *L. hesperus* herbivory.

 Thus, three forms of evidence, simulation modeling, manipulative experimentation, and the observational study that we report here, support the same inference. Cotton plants appear to have a window of heightened sensitivity to *L. hesperus* herbivory expressed early during the fruiting period. 

### Unnecessary use of insecticides

**Figure 4:**
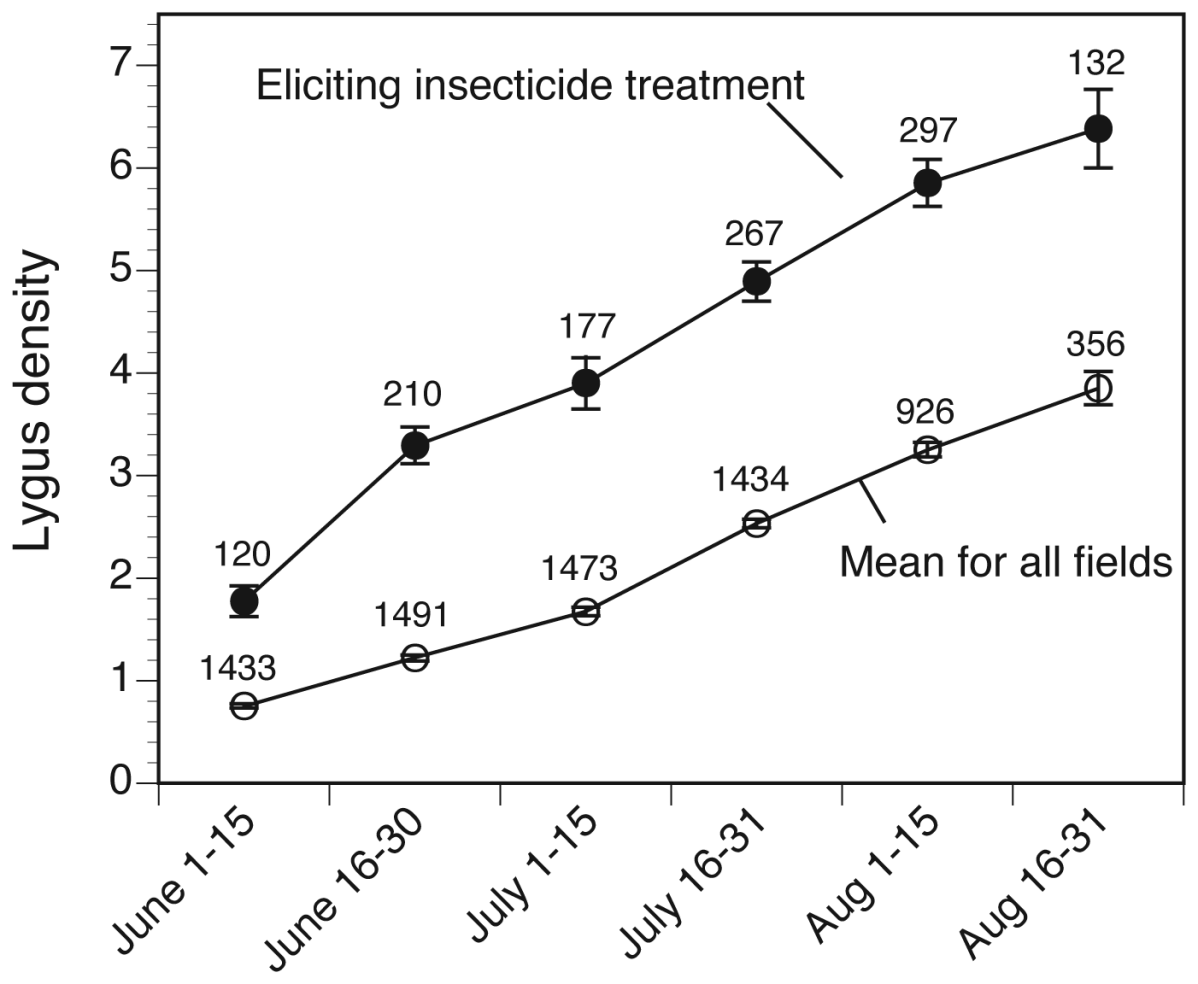
Insecticide use decisions and *L*. *hesperus* density. Mean ± SE density of *L*. *hesperus* that triggered the application of insecticides targeting *L*. *hesperus* (solid circles) during successive 2-week intervals during cotton’s fruiting period. Also shown for comparison are the mean *L*. *hesperus* densities observed across all fields (open circles). Numbers above the symbols are the number of fields for which density estimates were available.

Our analyses also identified conditions under which farmers appear to be applying insecticides unnecessarily to suppress economically benign *L. hesperus* populations. *L. hesperus* populations after mid-July do not appear to be associated with a detectable loss of yield. The mean density of *L. hesperus* that triggered the application of insecticides during late July was 4-5 bugs per sweep sample (Figure 4), comfortably within the range of densities that showed no effect on cotton yield (Figure 2B). Now that the early window of crop susceptibility has been identified, the hope is that farmers will become more receptive to the message that, according to their own data, cotton can compensate for moderate densities of *L. hesperus* later, after the sensitive period closes. 

Why might the sensitivity of cotton to *L. hesperus* decrease as the plant grows? The fruit maturation process in cotton appears to shift from being sink-limited to source-limited as the plant grows [15,18,23,35,36]. Young cotton plants have well-developed resource gathering structures (leaves and roots), but few flower buds. In this case, plants protected from herbivory or other stresses can retain and mature nearly 100% of flower buds initiated, and any flower bud abscission in response to *L. hesperus* feeding may translate into an immediate loss of yield (Figure 2A). In contrast, maturing plants rapidly produce a large number of flower buds, whose demands increasingly outstrip the plant’s resource supply. As a result, even in the absence of any environmental stress, abscission of flower buds or young fruit rapidly increases beginning around the time of flowering [18,23,35-37]. In this case, we may expect little effect of *L. hesperus* on yield as long as the proportion of flower buds damaged does not exceed that which the plant would abscise anyway [18]. Thus, the relationship between *L. hesperus* densities during July and cotton yield may be non-linear: an initial range of *L. hesperus* densities that generate no effect on yield is expected, as was found (Figure 2B). A subsequent phase of yield decline is, however, also expected as *L. hesperus* damages a proportion of flower buds that is greater than the proportion that would have been abscised in the absence of damage. Experiments suggest that this threshold is reached at densities of ca. 10 *L. hesperus* per sweep sample. 

This highlights an important limitation of the ecoinformatics approach: because farmers manage *L. hesperus* with insecticides in a universally aggressive manner, *L. hesperus* densities >10 per sample were never observed, even in a sample of >1000 commercial cotton fields (Figure 2). Thus, these farmer-derived data cannot be used to refine the critical late July *L. hesperus* density threshold at which yield loss begins. Observational research approaches like the one described here can only work with pre-existing variation. Thus, observational studies cannot explore scenarios like very high *L. hesperus* densities if, as was observed here, farmers exclude them the commercial setting. Experimentation has an obvious and important advantage in this regard.

A model of cotton yield that includes not only *L. hesperus* densities but also the number of insecticide applications targeting *L. hesperus* suggests that some additional yield loss (29.6 ± 10.8 kg/ha) is associated with each insecticide application, above and beyond the effects of *L. hesperus* herbivory (Table S3). This additional yield loss may reflect the disruptive effects of insecticide use on natural enemies, which can precipitate damaging outbreaks of other pests [30]. Thus, some July insecticide applications targeting *L. hesperus* may be not only unnecessary and costly, but even counterproductive. 

### Plant compensation by extending the growing season

The analysis presented here suggests that cotton is sensitive to very low densities of *L. hesperus* pre-flowering. Why couldn’t cotton compensate by developing additional fruit later in the growing season? Compensation ability is often treated as a plant trait, but the termination of crop growth is partially a response of the crop plant and partially a decision made by the farmer. Farmers have potent disincentives for delaying crop harvest, including the risk of fall rain, which degrades lint quality and can prevent harvest; the risk that late-season aphid or whitefly populations will build, producing honeydew that contaminates lint and reduces crop marketability; the cost of extra irrigation water needed to extend crop growth; and the pressing need to prepare fields for the following crop before rains arrive. For these reasons, an ecoinformatics approach that captures not just plasticity in plant growth, but also flexible farmer decision making is essential to measuring actual opportunities for plant compensation through delayed crop termination. Farmers delayed the termination of crops that harbored high *L. hesperus* populations during early July, but no such association was observed with June *L. hesperus* populations, perhaps contributing to the early window of crop sensitivity (Figure 3).

## Conclusions

Decentralized data collection in the agricultural sector can generate large datasets that hold the promise of greater statistical power. A proof of concept for an ecoinformatics approach focused on *L. hesperus* interactions with cotton suggested that the quality of consultant-generated data, as assessed by quantifying the relationship between *L. hesperus* and flower bud retention, was comparable to that of researcher-generated data. Strengths of the approach included (i) the ability to resolve small, but economically important yield gaps associated with an early window of crop sensitivity, and (ii) an opportunity to capture both plant and farmer responses to *L. hesperus* herbivory. A key weakness of the approach was the inability to consider the effects of very high *L. hesperus* densities, as these are excluded from the commercial farming setting by uniformly aggressive farmer pest suppression. The use of ecoinformatics in several subdisciplines of the agricultural sciences is growing [38-43], providing a research methodology that can complement traditional experimental approaches to enhancing agricultural productivity. 

## Supporting Information

Figure S1 **Association between the cotton yield gap (difference between the observed and expected yield, after controlling for annual fluctuations in valley-wide yield) and mean *L. hesperus* densities observed during (**A**) 1-15 June, (**B**) 16-30 June, (**C**) 1-15 July, and (**D**) 16-31 July.** The solid lines are the smooths from the GAM models, and the shaded regions are the 95% confidence intervals. Note that because the GAM penalizes the second derivative of the fitted curve, the model collapses to linearity when evidence for non-linearity is sufficiently weak, as is seen in panels (A), (B), and (D).
(EPS)

Table S1 **Generalized additive model of factors associated with yield of cotton, *Gossypium* spp., with the fruiting season broken into successive 2-week intervals.**
(DOCX)

Table S2 **Generalized additive model of factors associated with the date of the first application of defoliant to cotton, *Gossypium* spp., including both *L. hesperus* densities and the monthly mean temperatures and rainfall beginning October of the year preceding the spring planting of the focal cotton crop.**
(DOCX)

Table S3 **Generalized additive model of factors associated with yield of cotton, *Gossypium* spp., including both *L. hesperus* densities and the number of insecticide applications that targeted *L. hesperus*.**
(DOCX)

Table S4 **Generalized additive model of factors associated with yield of cotton, *Gossypium* spp., including both *L. hesperus* densities and planting date.**
(DOCX)

Table S5 **Generalized additive model of factors associated with yield of cotton, *Gossypium* spp., including both *L. hesperus* densities and field size.**
(DOCX)

Table S6 **Generalized additive model of factors associated with yield of cotton, *Gossypium* spp., including *L. hesperus* densities and the number of insecticide applications made targeting spider mites (*Tetranychus* spp.), aphids (*Aphis gossypii*), whiteflies (*Bemisia tabaci* and *Trialeurodes vaporariorum*), thrips (mostly *Frankliniella occidentalis*), and various Lepidoptera (*Spodoptera* spp. and others).**
(DOCX)

Table S7 **Generalized additive model of factors associated with yield of cotton, *Gossypium* spp., including both *L. hesperus* densities and the numbers of applications of either plant growth regulators or defoliants.**
(DOCX)

Table S8 **Generalized additive model of factors associated with yield of cotton, *Gossypium* spp., including both *L. hesperus* densities and the monthly mean temperatures and rainfall beginning October of the year preceding the spring planting of the focal cotton crop.**
(DOCX)
