# Supplementary material for: Ecoinformatics Can Reveal Yield Gaps Associated with Crop-Pest Interactions: A Proof-of-Concept
Source: PLoS One. 2013 Nov 15;8(11):e80518. doi: 10.1371/journal.pone.0080518 (PMC3829906; doi:10.1371/journal.pone.0080518)
Supplement: Table S1 — Generalized additive model of factors associated with yield of cotton, Gossypium spp., with the fruiting season broken into successive 2-week intervals. (DOCX) [file pone.0080518.s002.docx]

Table S1. Generalized additive model of factors associated with yield of cotton, *Gossypium* spp., with the fruiting season broken into successive 2-week intervals

| Term | df | *F* | *P* |
| --- | --- | --- | --- |
| Farm | 35 | 2.40 | 1.3x10^-5^ |
| Year | 10 | 12.57 | <1x10^-15^ |
| *Gossypium* species | 1 | 0.04 | 0.84 |
| 1-15 June *L. hesperus* density | 1 | 13.80 | 0.0002 |
| 16-30 June *L. hesperus* density | 1 | 6.62 | 0.010 |
| 1-15 July *L. hesperus* density | 7.49 | 2.00 | 0.041 |
| 16-31 July *L. hesperus* density | 1 | 2.72 | 0.099 |

Deviance explained = 21.8%, *N* = 1052
