# Supplementary material for: Ecoinformatics Can Reveal Yield Gaps Associated with Crop-Pest Interactions: A Proof-of-Concept
Source: PLoS One. 2013 Nov 15;8(11):e80518. doi: 10.1371/journal.pone.0080518 (PMC3829906; doi:10.1371/journal.pone.0080518)
Supplement: Table S2 — Generalized additive model of factors associated with the date of the first application of defoliant to cotton, Gossypium spp., including both L. hesperus densities and the monthly mean temperatures and rainfall beginning October of the year preceding the spring planting of the focal cotton crop. (DOCX) [file pone.0080518.s003.docx]

Table S2. Generalized additive model of factors associated with the date of the first application of defoliant to cotton, *Gossypium* spp., including both *L. hesperus* densities and the monthly mean temperatures and rainfall beginning October of the year preceding the spring planting of the focal cotton crop

| Term | df | *F* | *P* |
| --- | --- | --- | --- |
| Farm | 37 | 7.16 | <1x10^-15^ |
| Year | 11 | 11.05 | 0.00092 |
| *Gossypium* species | 1 | 150.19 | <1x10^-15^ |
| October mean temperature | 1 | 0.23 | 0.635 |
| November mean temperature | 1 | 0.06 | 0.812 |
| December mean temperature | 1 | 23.87 | 1.17x10^-6^ |
| January mean temperature | 1 | 7.75 | 0.0055 |
| February mean temperature | 1 | 4.09 | 0.043 |
| March mean temperature | 1 | 65.56 | 1.38x10^-15^ |
| April mean temperature | 1 | 5.90 | 0.015 |
| May mean temperature | 1 | 2.85 | 0.092 |
| June mean temperature | 1 | 13.45 | 0.00026 |
| July mean temperature | 1 | 32.29 | 1.67x10^-8^ |
| August mean temperature | 1 | 65.38 | 1.50x10^-15^ |
| September mean temperature | 1 | 34.32 | 6.05x10^-9^ |
| October rain | 1 | 0.51 | 0.475 |
| November rain | 1 | 15.29 | 9.76x10^-5^ |
| December rain | 1 | 12.53 | 0.00042 |
| January rain | 1 | 0.032 | 0.857 |
| February rain | 1 | 25.36 | 5.50x10^-7^ |
| March rain | 1 | 3.52 | 0.061 |
| April rain | 1 | 0.09 | 0.770 |
| May rain | 1 | 0.19 | 0.662 |
| June rain | 1 | 39.34 | 4.97x10^-10^ |
| 1-15 June *L. hesperus* density | 1.63 | 0.60 | 0.556 |
| 16-30 June *L. hesperus* density | 1 | 2.54 | 0.111 |
| 1-15 July *L. hesperus* density | 4.29 | 4.49 | 0.00033 |
| 16-31 July *L. hesperus* density | 2.23 | 1.72 | 0.163 |

Deviance explained = 43.9%, *N* = 1265
