# Supplementary material for: Ecoinformatics Can Reveal Yield Gaps Associated with Crop-Pest Interactions: A Proof-of-Concept
Source: PLoS One. 2013 Nov 15;8(11):e80518. doi: 10.1371/journal.pone.0080518 (PMC3829906; doi:10.1371/journal.pone.0080518)
Supplement: Table S3 — Generalized additive model of factors associated with yield of cotton, Gossypium spp., including both L. hesperus densities and the number of insecticide applications that targeted L. hesperus. (DOCX) [file pone.0080518.s004.docx]

Table S3. Generalized additive model of factors associated with yield of cotton, *Gossypium* spp., including both *L. hesperus* densities and the number of insecticide applications that targeted *L. hesperus*

| Term | df | *F* | *P* |
| --- | --- | --- | --- |
| Farm | 35 | 2.51 | 4.1x10^-6^ |
| Year | 10 | 12.36 | <1x10^-15^ |
| *Gossypium* species | 1 | 0.18 | 0.67 |
| Insecticides targeting *L. hesperus* | 1 | 7.53 | 0.006 |
| June *L. hesperus* density | 6.13 | 4.57 | 3.5x10^-5^ |
| July *L. hesperus* density | 2.82 | 1.43 | 0.23 |

Deviance explained = 23.2%, *N* = 1106

In a multiple regression model including main effects for Farm, Year, and *Gossypium* species using the full data set, the number of insecticide applications targeting *Lygus* was positively correlated with *L. hesperus* densities during both June (*F* = 822.8, *N* = 1467, *P* < 0.0001) and July (*F* = 282.1, *N* = 1450, *P* < 0.0001). Nevertheless, inclusion of the number of applications in the model did not generate a qualitative change in the relationship between *Lygus* densities and cotton yield.
