# Supplementary material for: Ecoinformatics Can Reveal Yield Gaps Associated with Crop-Pest Interactions: A Proof-of-Concept
Source: PLoS One. 2013 Nov 15;8(11):e80518. doi: 10.1371/journal.pone.0080518 (PMC3829906; doi:10.1371/journal.pone.0080518)
Supplement: Table S4 — Generalized additive model of factors associated with yield of cotton, Gossypium spp., including both L. hesperus densities and planting date. (DOCX) [file pone.0080518.s005.docx]

Table S4. Generalized additive model of factors associated with yield of cotton, *Gossypium* spp., including both *L. hesperus* densities and planting date

| Term | df | *F* | *P* |
| --- | --- | --- | --- |
| Farm | 24 | 3.16 | 8.0x10^-7^ |
| Year | 10 | 10.29 | <1x10^-15^ |
| *Gossypium* species | 1 | 0.65 | 0.42 |
| Planting date | 1 | 6.25 | 0.013 |
| June *L. hesperus* density | 1 | 16.61 | 5.2x10^-5^ |
| July *L. hesperus* density | 3.47 | 1.46 | 0.21 |

Deviance explained = 24.9%, *N* = 684

Earlier planting was associated with a modest increase yield (yield increased by 4.52 ± 1.81 kg/ha per day that planting was advanced). In a multiple regression model including main effects for Farm, Year, and *Gossypium* species using the full data set, Planting date was not significantly correlated with *L. hesperus* densities during either June (*F* = 1.16, *N* = 847, *P* = 0.06) or July (*F* = 0.10, *N* = 847, *P* = 0.75).
