# Supplementary material for: Ecoinformatics Can Reveal Yield Gaps Associated with Crop-Pest Interactions: A Proof-of-Concept
Source: PLoS One. 2013 Nov 15;8(11):e80518. doi: 10.1371/journal.pone.0080518 (PMC3829906; doi:10.1371/journal.pone.0080518)
Supplement: Table S5 — Generalized additive model of factors associated with yield of cotton, Gossypium spp., including both L. hesperus densities and field size. (DOCX) [file pone.0080518.s006.docx]

Table S5. Generalized additive model of factors associated with yield of cotton, *Gossypium* spp., including both *L. hesperus* densities and field size

| Term | df | *F* | *P* |
| --- | --- | --- | --- |
| Farm | 35 | 2.43 | 9.2x10^-6^ |
| Year | 10 | 12.55 | <1x10^-15^ |
| *Gossypium* species | 1 | 0.10 | 0.76 |
| Field size | 1 | 0.00 | 0.98 |
| June *L. hesperus* density | 6.39 | 9.23 | 7.9x10^-12^ |
| July *L. hesperus* density | 2.85 | 0.77 | 0.53 |

Deviance explained = 22.0%, *N* = 1118

In a multiple regression model including main effects for Farm, Year, and *Gossypium* species using the full data set, field size was not significantly correlated with *L. hesperus* densities during either June (*F* = 1.63, *N* = 1433, *P* = 0.20) or July (*F* = 2.60, *N* = 1433, *P* = 0.11). Inclusion of Field size in the model had a negligible influence on the relationship between *Lygus* densities and cotton yield.
