# Supplementary material for: Ecoinformatics Can Reveal Yield Gaps Associated with Crop-Pest Interactions: A Proof-of-Concept
Source: PLoS One. 2013 Nov 15;8(11):e80518. doi: 10.1371/journal.pone.0080518 (PMC3829906; doi:10.1371/journal.pone.0080518)
Supplement: Table S6 — Generalized additive model of factors associated with yield of cotton, Gossypium spp., including L. hesperus densities and the number of insecticide applications made targeting spider mites (Tetranychus spp.), aphids (Aphis gossypii), whiteflies (Bemisia tabaci and Trialeurodes vaporariorum), thrips (mostly Frankliniella occidentalis), and various Lepidoptera (Spodoptera spp. and others). (DOCX) [file pone.0080518.s007.docx]

Table S6. Generalized additive model of factors associated with yield of cotton, *Gossypium* spp., including *L. hesperus* densities and the number of insecticide applications made targeting spider mites (*Tetranychus* spp.), aphids (*Aphis gossypii*), whiteflies (*Bemisia tabaci* and *Trialeurodes vaporariorum*), thrips (mostly *Frankliniella occidentalis*), and various Lepidoptera (*Spodoptera* spp. and others)

| Term | df | *F* | *P* |
| --- | --- | --- | --- |
| Farm | 35 | 2.43 | 9.2x10^-6^ |
| Year | 10 | 12.55 | <1x10^-15^ |
| *Gossypium* species | 1 | 0.10 | 0.76 |
| Field size | 1 | 0.00 | 0.98 |
| Spider mites | 1 | 0.19 | 0.66 |
| Aphids | 1 | 0.31 | 0.58 |
| Whiteflies | 1 | 0.18 | 0.67 |
| Thrips | 1 | 1.92 | 0.17 |
| Lepidoptera | 1 | 0.32 | 0.57 |
| June *L. hesperus* density | 6.43 | 9.53 | 2.6x10^-12^ |
| July *L. hesperus* density | 2.76 | 1.05 | 0.38 |

Deviance explained = 22.9%, *N* = 1106

In a multiple regression model including main effects for Farm, Year, and *Gossypium* species using the full data set, some associations were found between insecticide use and observed *L. hesperus* densities during June (significant negative relationships for the number of applications targeting spider mites [*F* = 7.32, *P* = 0.0069] and Lepidoptera [*F* = 5.98, *P* = 0.015], a significant positive relationship with applications targeting thrips [*F* = 18.1, *P* < 0.0001], and non-significant relationships for applications targeting aphids and whiteflies [*P* > 0.7 for each], *N* = 1467). Only the number of insecticide applications targeting thrips was significantly correlated with July *Lygus* densities (*F* = 8.10, *N* = 1450, *P* = 0.0045; all other variables were not significantly correlated, *P* > 0.10). Inclusion of insecticide use variables into the GAM analysis did not change the underlying relationship between *Lygus* densities and cotton yield.
