# Supplementary material for: Ecoinformatics Can Reveal Yield Gaps Associated with Crop-Pest Interactions: A Proof-of-Concept
Source: PLoS One. 2013 Nov 15;8(11):e80518. doi: 10.1371/journal.pone.0080518 (PMC3829906; doi:10.1371/journal.pone.0080518)
Supplement: Table S7 — Generalized additive model of factors associated with yield of cotton, Gossypium spp., including both L. hesperus densities and the numbers of applications of either plant growth regulators or defoliants. (DOCX) [file pone.0080518.s008.docx]

Table S7. Generalized additive model of factors associated with yield of cotton, *Gossypium* spp., including both *L. hesperus* densities and the numbers of applications of either plant growth regulators or defoliants

| Term | df | *F* | *P* |
| --- | --- | --- | --- |
| Farm | 35 | 2.36 | 1.9x10^-5^ |
| Year | 10 | 13.35 | <1x10^-15^ |
| *Gossypium* species | 1 | 0.04 | 0.84 |
| Plant growth regulator applications | 1 | 1.16 | 0.28 |
| Defoliant applications | 1 | 2.37 | 0.12 |
| June *L. hesperus* density | 6.23 | 9.41 | 7.0x10^-12^ |
| July *L. hesperus* density | 2.72 | 0.65 | 0.61 |

Deviance explained = 22.9%, *N* = 1106
